# Diagnosis and treatment of microsporidial keratoconjunctivitis: literature review and case series

**DOI:** 10.1007/s12348-011-0025-y

**Published:** 2011-05-11

**Authors:** Sadik Taju, Yonas Tilahun, Menen Ayalew, Nigus Fikrie, Jakob Schneider, John H. Kempen

**Affiliations:** 1 Department of Ophthalmology, Addis Ababa University, Addis Ababa, Ethiopia; 2 Department of Microbiology and Parasitology, Addis Ababa University, Addis Ababa, Ethiopia; 3 Department of Pathology, Addis Ababa University, Addis Ababa, Ethiopia; 4 Departments of Ophthalmology and Biostatistics & Epidemiology and the Center for Clinical Epidemiology and Biostatistics, University of Pennsylvania School of Medicine, Philadelphia, PA USA

**Keywords:** Microsporidia, Keratoconjunctivitis, HIV/AIDS, Light microscopy, Albendazole

## Abstract

**Purpose:**

The purpose of this study is to describe the clinical characteristics, microscopic findings, and treatment response to albendazole of microsporidial keratoconjunctivitis among immunocompromised individuals with HIV/AIDS.

**Methods:**

This is a retrospective case series. Diagnosis of microsporidial keratoconjunctivitis was confirmed by subspecialist examination and conjunctival swabs examined by light microscopy. HIV infection was documented, and absolute CD4+ T cell count was determined. Patients were treated with albendazole and followed for clinical response.

**Results:**

Light microscopy from the conjunctival swabs demonstrated myriad small, round to oval microsporidial organisms that stained positively with modified acid-fast methods. Two of the patients initially not taking highly active antiretroviral therapy (HAART) and presenting with an absolute CD4+ T cell count less than 100 cells/μL had a more severe form of keratoconjunctivitis than the third patient (receiving HAART, with a CD4+ T cell count of 259 cells/μL). All patients were started or continued on HAART. Two of the patients responded to oral albendazole, with resolution of symptoms and signs. The third patient did not initially respond, perhaps because of an immune recovery inflammatory syndrome, but subsequently had temporary improvement with albendazole.

**Conclusions:**

Microsporidial keratoconjunctivitis is a rare ocular complication of HIV/AIDS. Light microscopic evaluation of conjunctival swabs may be a useful minimally invasive first step toward diagnosis of microsporidial keratoconjunctivis in settings where electron microscopy is not available. Based on the limited available information, albendazole often is effective for this condition, and is widely available in developing countries at low cost.

## Introduction and literature review

Microsporidia are obligate intracellular spore-forming eukaryotic protozoan parasites, some of which are pathogenic in humans [1]. They are ubiquitous in the environment, and can infect a wide range of vertebrate and invertebrate hosts, including insects, birds, fish, and mammals [2]. To date, up to 150 genera containing over 1,200 species have been described. Of these genera, six are known to infect human beings: *Encephalitozoon*, *Enterocytozoon*, *Nosema*, *Pleistophora*, and *Trachipleistophora* [3]. The spore is resistant to degradation, surviving in the environment for up to 4 months [4] in its infective form. Humans acquire infection through ingestion, inhalation, or sexual transmission of spores [4]. Although reliable prevalence data are lacking, recent studies indicate increased risk of microsporidial keratoconjunctivitis in the rainy season [5].

It is not clear how microsporidia enter into the cornea; traumatic inoculation or contact with contaminated water or food has been suggested [4]. Once invasion of the spore into the human host cell occurs, the contents are discharged into the cytoplasm. Within the cell, the sporoplast divides by binary fission to form a schizont with two to six nuclei, which split into unicellular meronts. The meronts then secrete a rigid capsule; the fully formed spore measures about 2.5 × 1.5 μm. The cell eventually ruptures to continue the cycle of further destruction of the host tissue [3].

Clinical symptoms and disease associated with microsporidiosis vary with the species that causes the infection and the host’s immune status [6]. Most cases of intestinal and disseminated microsporidiosis in patients infected with HIV are reported in patients who are severely immunocompromised (CD4+ T cell count <100/mm^3^); in these patients, morbidity can be significant [7].

Microsporidial infections of the ocular surface in humans are rare diseases. Microsporidial keratoconjunctivitis has been described primarily in patients with HIV infection [6], but also has been reported in normal hosts [5, 8]. There are two main clinical presentations of ocular microsporidiosis: a necrotizing stromal keratitis that occurs in immunocompetent individuals and an epithelial keratoconjunctivitis described in HIV-infected individuals [3, 6]. Bilateral punctuate epithelial involvement of the cornea with white intraepithelial infiltrates that stain irregularly with fluorescein is the typical pattern of ocular microsporidiosis described in immunocompromised individuals [6]. Deep stromal disease may or may not be present. Other findings include a mild anterior chamber reaction and a mild conjunctivitis. Presenting symptoms include foreign body sensation, dryness, redness, reduced visual acuity, and photophobia. Ocular surface disease appears to be less severe when CD4+ T cell counts are low; with immune reconstitution, signs and symptoms may become more pronounced [6]. Although primarily described in patients with AIDS, a pattern of ocular microsporidiosis with minimal inflammatory signs also has been reported to occur in immunocompetent individuals receiving topical corticosteroid therapy [3–7].

Diagnosis of ocular microsporidiosis is based on scrapings or biopsy of the conjunctiva or cornea. Pathologically, microsporidial spores are ovoid and may be found in the cytoplasm of the epithelial cells, sometimes measuring only 2 × 1 μm. There will be few accompanying inflammatory cells in individuals with pronounced immunodeficiency. The organisms are Gram positive and stain well with Giemsa stain; characteristic periodic acid–Schiff-positive granules are found in one end of the mature microsporidial spores. Diagnosis may be aided by the use of electron microscopy and confocal microscopy in vivo [8, 9], sophisticated, expensive technologies. It is difficult to culture these organisms from clinical specimens, and reliable serologic tests are not available for the diagnosis of microsporidiosis [8, 9]. Transmission electron microscopy has been the gold standard for definitive diagnosis of microsporidial infection; molecular methods (most making use of the polymerase chain reaction) have been developed as alternative approaches for the laboratory diagnosis of microsporidiosis [8]. These methods have not been available in developing countries such as Ethiopia.

The best treatment for microsporidial keratitis has not been established. Treatment with 0.02% polyhexamethylene biguanide does not offer any significant advantage over placebo [5]. Microsporidial infections in HIV-infected individuals may respond to combination of antibiotics and antiparasitic agents, including topical propamidine isethionate, topical fumagillin, topical fluoroquinolones, oral albendazole, and/or oral itraconazole. However, treatment often has been followed by recrudescence of infection [1, 4, 7, 10]. One report describes unmasking of microsporidiosis infection following HAART-induced immune recovery [11], likely representing an immune recovery inflammatory syndrome [12]. Topical steroids in immune-competent patients with microsporidiosis seem to contribute to the persistence of infection and may be a predisposing factor in these cases by creating a localized immunocompromised state [13].

Although one case has been reported in a patient who had lived in Mozambique [14], to the best of our knowledge, no cases have been reported from centers in Africa to date. In order to better characterize the nature of this condition, in addition to the previous review of the available literature on the subject of microsporidial keratitis, we now report the clinical and pathological characteristics and the outcomes of a cluster of three additional cases presenting for care in Addis Ababa, Ethiopia during a 5-month period in 2008.

## Methods

We retrospectively reviewed the clinical features, microscopic observations, and treatment response to albendazole of a cluster of immune-compromised patients with keratoconjunctival microsporidiosis seen at the anterior segment clinic of Menelik II Hospital, the primary cornea and external diseases center for the Department of Ophthalmology, Addis Ababa University. In each case, best-corrected visual acuity had been measured at 6 m for each eye of each patient. Clinical findings were based on slit-lamp biomicroscopy by a consultant cornea specialist.

Specimens were obtained and evaluated by the Department of Microbiology and Parasitology of Black Lion Hospital, Addis Ababa University. Conjunctival swabs, as a minimally invasive first step toward diagnosis, were collected from each patient suspected of having microsporidiosis and directly smeared on a slide and allowed to air-dry prior to fixation with methanol. The slides were stained with the modified trichrome stain solution for 90 min and then rinsed for 10 s with acid alcohol, rinsed briefly with 95% ethanol, and incubated for 5 min in 95% ethanol and for 10 min in 100% ethanol. The slides were air-dried and examined microscopically with oil immersion. The microsporidial spores stain dark violet against a pale-green background.

After confirmation of microsporidial keratitis, CD4+ T cell counts were determined, and highly active antiretroviral therapy (HAART) initiated or continued. For patients who failed to improve on HAART alone, either one or two courses of albendazole was initiated, as alternative treatments were not available in Ethiopia at the time of the study. Patients’ response to the treatment was evaluated clinically following treatment.

## Results

Among over 3,000 patients seen in the cornea and external diseases clinic between June 2008 and November 2008 inclusive, three cases of microsporidial infection were identified, all in patients with HIV/AIDS.

### Case 1

**Fig. 1:**
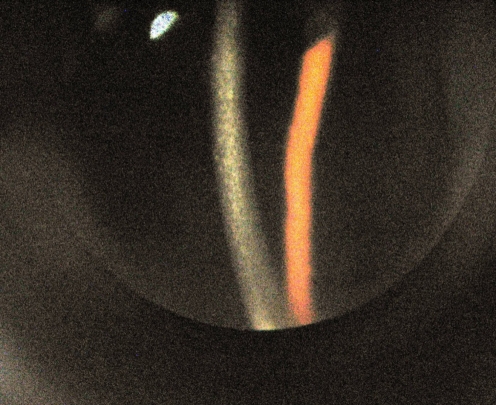
Slit-lamp photograph showing the slit-lamp findings of case 1 (intraepithelial corneal infiltrates in a case of microsporidial keratoconjunctivitis)

A 10-year-old female patient from Addis Ababa, discovered to be HIV seropositive a year previously, presented with 3 months history of redness, tearing, photophobia, and reduction of vision of both eyes. Her best-corrected visual acuity was 6/60 (6/24 with pin hole) and counting fingers at 4 m (no improvement with pin hole) in the right and left eyes, respectively. She had a healed zoster scar over the left side of the forehead. The conjunctivae were hyperemic with ciliary flush. Corneal sensation was diminished bilaterally, and diffuse epithelial and subepithelial whitish corneal infiltrates were present (Fig. 1), which stained with fluorescein.

**Fig. 2:**
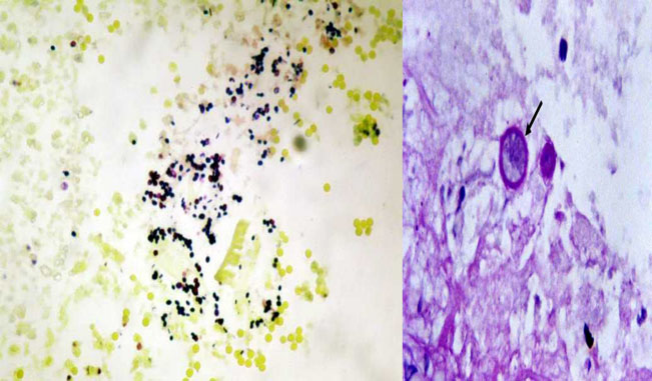
Microsporidial spores staining dark violet against a pale-green background in a conjunctival swab specimen from case 3. Microsporidial spores (*large arrow*) are seen as well-defined oval reddish bodies with a dark staining of the narrow end of the spore (*black*) or a waistband (*white*) closer to the tip of narrow end. Also seen are the unstained blue spores (*small arrow*) which possibly are immature or degenerating spores (1% acid-fast stain, ×1,000)

Prior to her presentation, treatment with gentamicin eye drops had been followed by marked worsening of visual acuity, redness, tearing, and photophobia. After initial treatment with artificial tears, chloramphenicol eye ointment, acyclovir, and topical corticosteroids failed to improve her condition, a conjunctival swab was taken from the patient for microscopic investigation, and the CD4+ T cell count was determined. Light microscopy demonstrated myriad small, round to oval microsporidial organisms that stained positively with modified acid-fast methods (see Fig. 2, left panel). Based on these findings, and a CD4+ T cell count of 71 cells/μL, she was started on HAART.

One month after initiating the HAART, the patient continued to have symptoms of bilateral foreign body sensation, redness, tearing, and blurred vision, and examination findings were unchanged. A course of oral albendazole 200 mg daily for 14 days was initiated. Following completion of the course, symptoms of redness, foreign body sensation, and photophobia were markedly reduced. The visual acuity improved to 6/9 and 6/12 in right and left eyes respectively, the conjunctivae were white and quiet, and only scanty intraepithelial white infiltrates remained in the peripheral corneas. During monthly follow-up over the next 3 months, no recurrence was observed; the CD4 count was found to have increased to 189 cells/μL by that time.

### Case 2

A married 40-year-old female from Addis Ababa presented with a 1-year history of unilateral (left eye) redness, episodic photophobia, and blurring of vision. She gave history of having had loss of vision in the right eye following severe redness, pain, photophobia, and excessive tearing 2 years prior to her presentation. She denied a previous history of contact with animals or agricultural trauma. She was known to be HIV positive, having taken HAART for the past 4 years, with a recent CD4+ T cell count of 259 cells/μL. She had not been taking topical therapies at the time of presentation.

On examination, the visual acuity of the left eye was 6/9. The bulbar conjunctiva was injected nasally, and the cornea had yellowish stromal and intraepithelial infiltrates that stained with fluorescein, although there was no epithelial defect (findings similar to those illustrated in Fig. 1). A microscopic examination of a conjunctival swab using light microscopy demonstrated myriad small, round to oval microsporidial organisms that stained positively with modified acid-fast methods, confirming microsporidial keratitis (findings similar to those illustrated in Fig. 2). After 2 weeks of treatment with albendazole 400 mg twice daily, while continuing HAART, all the ocular complaints disappeared, and the corneal opacity was less dense as compared to prior to initiating the albendazole treatment. Similar to the first case, the patient had no recurrence of signs or symptoms over 3 months follow-up.

### Case 3

A 24-year-old woman from Addis Ababa presented with a 7-month history of bilateral foreign body sensation, redness, tearing, and reduction in vision, which had not improved with 1 month of topical corticosteroids. She was known to be HIV positive, but had not received HAART previously. She had no history of contact with animals or agricultural trauma. At initial presentation, her visual acuity was 6/24 and 6/9 on the right and left eyes, respectively. In each eye, the conjunctivae were hyperaemic, and there were diffuse intraepithelial whitish infiltrates over the cornea with punctate fluorescein staining. Light microscopy of a conjunctival swab revealed myriad small, round to oval microsporidial organisms that stained positively with modified acid-fast method for microsporidia.

**Fig. 3:**
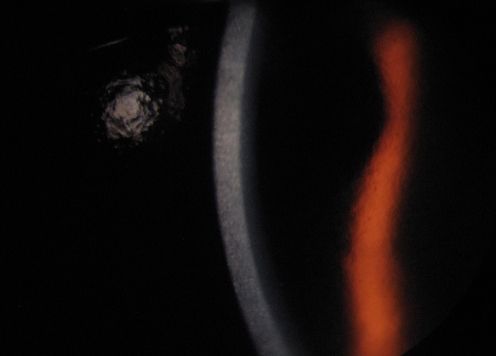
Slit-lamp photograph showing the slit-lamp findings of case 3 (intraepithelial corneal infiltrates in a case of microsporidial keratoconjunctivitis)

After the CD4+ T cell count was found to be 45 cells/μL, HAART was initiated 1 day after taking the conjunctival swab. One month after HAART, there was little symptomatic or objective improvement in the ophthalmic findings, and the CD4+ T cell count had improved to 179 cells/μL. Thereafter, she was treated with albendazole 400 mg twice daily for 2 weeks. At the second month, she presented with worsening of redness and severe foreign body sensation and no visual improvement; she developed a corneal epithelial defect over the left eye, and the preexisting intraepithelial lesions had become more flocculent in appearance (Fig. 3). Although the corneal epithelial defect healed after patching, the clinical findings failed to resolve after a second course of albendazole, unlike the other two cases. However, the symptoms and signs eventually subsided. On suspicion that the continued inflammation despite albendazole was the result of an immune recovery inflammatory syndrome, additional 2-week courses of albendazole were attempted, after which symptoms improved but relapsed 3–4 months after each course.

## Discussion

The first case of microsporidiosis in an HIV-infected individual was reported in 1985 [1]; since then, over 400 cases have been reported. Microsporidiosis appears to occur most commonly in severely immunodeficient individuals, especially patients having CD4 + T-lymphocyte counts <100 cells/μL [7, 12, 15], although it is a rare condition even among these individuals. As in our cases, microsporidiosis consistently has been reported as limited to the corneal epithelium in case reports from immunodeficient patients [3, 6].

Although the second case was on HAART for 4 years before presentation, the other two cases presented with a CD4+ T cell count <100 cells/μL. We hypothesize that the lesser degree of ocular discomfort and less dramatic physical findings in the second patient might have been related to her successful treatment with antiretroviral therapy, although a small series such as this one is unable to confirm such a hypothesis. If so, early diagnosis of HIV infection and initiation of HAART may improve the clinical course of microsporidial keratoconjunctivitis in some cases, in addition to the numerous other benefits of such therapy for patients with advanced HIV/AIDS [11, 15]. Such benefits most likely would outweigh the potential to develop an immune recovery inflammatory syndrome in occasional cases [12], such as may have happened in patient 3 2 months after starting HAART.

Detection of microsporidiosis classically has relied on electron microscopy and the histopathology of corneal or conjunctival biopsy specimens, the latter being essential for accurate speciation. In our report the microsporidial spores were identified using the simple, inexpensive approach of using modified acid-fast staining technique applied to a specimen taken from a conjunctival swab, a minimally invasive approach that is potentially an appropriate first step toward the diagnosis of microsporidiosis in settings with limited resources. While in our cases simple conjunctival swabs yielded the diagnosis, the approach likely would have lesser sensitivity and specificity than more invasive approaches; corneal scraping and/or conjunctival biopsy likely would be needed in some instances to make the diagnosis.

Therapeutically, there is no standard medical therapy for corneal microsporidiosis. However, the most frequently used approach has been topical fumagillin [15] and/or oral albendazole, which have been associated with improvement in many but not all cases [16, 17]. Topical fluoroquinolone monotherapy also has been reported as a useful treatment option for microsporidial keratitis [10]. There also have been reports of response of cases of keratoconjunctivitis to topical brolene [18] or oral itraconazole [19]. Our experience demonstrates successful treatment in two of three cases and what we interpret as partial success in the third using albendazole, an inexpensive treatment (less than US $1 per course in Ethiopia), which is widely available and suitable for use in resource-limited settings. Laboratory determination of microsporidial sensitivity potentially could identify alternative regimens for strains and alternate species which prove not to be sensitive to treatment with albendazole; sensitivity testing was not available in our setting, so we were unable to evaluate case 3 for resistance.

## Conclusion

Microsporidiosis should be considered in the differential diagnosis of chronic keratoconjunctivitis in immunodeficient patients. Reciprocally, patients with microsporidial keratoconjunctivitis should be evaluated for possible immunodeficiency syndromes such as HIV/AIDS, given the known association between these conditions, and the increasing availability of effective treatment for HIV/AIDS. Based on our small sample and the limited reported experience in the literature, albendazole, which is inexpensive and widely available, is a practical treatment approach when the diagnosis of microsporidial keratoconjunctivitis is recognized in a resource-constrained setting.
